# Immune-Enhancing Effects of *Polygonatum cyrtonema* Polysaccharides in Immunodeficient Zebrafish

**DOI:** 10.3390/cimb48050494

**Published:** 2026-05-09

**Authors:** Daoyuan Li, Jie Wang, Naifu Chen, Naidong Chen

**Affiliations:** 1 College of Life and Health, West Anhui University, Lu’an 237012, China; 789ldy123@163.com (D.L.); wj062199@163.com (J.W.); 2 Anhui Provincial Key Laboratory for Quality Evaluation and Improvement of Traditional Chinese Medicine, Lu’an 237012, China; 3 Anhui Provincial Engineering Research Center for Conservation and Utilization of Chinese Medicinal Resources, Lu’an 237012, China; 4 Experimental and Practical Training Teaching Administration Office, West Anhui University, Lu’an 237012, China

**Keywords:** *Polygonatum cyrtonema* polysaccharides, immunomodulation, zebrafish, neutrophils

## Abstract

To evaluate the immune-enhancing effects of *Polygonatum cyrtonema* polysaccharides in vivo, an immunodeficiency zebrafish model was established by microinjecting vinorelbine tartrate into the caudal vein. Effects of the polysaccharides (500, 1000 and 2000 μg/mL) on neutrophil counts were assessed in *Tg (mpx:GFP)* zebrafish. Transcriptome sequencing was employed to investigate the immunomodulatory effects of the polysaccharides. The results revealed a dose-dependent increase in neutrophil counts following treatment with the polysaccharides. Transcriptomic profiling identified 1286 DEGs across the three comparison groups. GO and KEGG enrichment analyses indicated that the polysaccharides could modulate immune-related pathways in the zebrafish model. Two enriched KEGG pathways, including the MAPK signaling and the mTOR signaling pathway, were utilized to analyze immune-related gene expression. To validate RNA-seq data, qRT-PCR was performed on selected DEGs, including *il1b*, *crk*, *fgf10b*, *atp6v1aa*, and *eif4e1c*. The results confirmed that the expression patterns of these genes were consistent with the RNA-seq data. Within the tested concentrations (500, 1000 and 2000 μg/mL), the polysaccharides exhibited a dose-dependent immunostimulatory effect, with the highest immunostimulatory response observed at 2000 μg/mL. The molecular level primarily involves the enhancement of neutrophil function through the modulation of multiple immune-related pathways. These findings provide a theoretical basis for the potential application of *Polygonatum cyrtonema* polysaccharides as a natural immunomodulatory agent.

## 1. Introduction

*Polygonatum cyrtonema* Hua, a member of the Liliaceae family, is widely distributed in Asia and regarded as one of the essential herbs in traditional Chinese medicine [1,2]. Its therapeutic properties are attributed to its abundant bioactive compounds, including polysaccharides, saponins, alkaloids, flavonoids, and lignanoids [1,3,4]. *Polygonatum cyrtonema* polysaccharides (PCPs) have been identified as the primary active components, exhibiting a broad spectrum of pharmacological activities such as immunomodulation, antioxidation, anti-diabetic effects, anti-inflammation, and anti-tumor properties [3,5].

Several studies have demonstrated that *Polygonatum* polysaccharides exhibit immunomodulatory activity. Na Liu et al. [1] reported that polysaccharides extracted from *Polygonatum sibiricum* can activate macrophages, enhance their phagocytic activity, promote splenocyte proliferation, and increase the thymus and spleen indices in immunosuppressed mice. *Polygonatum* polysaccharides also have been reported to stimulate the proliferation of T and B lymphocytes and enhance the phagocytic activity of peritoneal macrophages, indicating their potent immunoregulatory effects [1,6]. Similarly, another study demonstrated that *Polygonatum* polysaccharides possess immunomodulatory properties, characterized by their ability to enhance cytokine secretion and antioxidant enzyme activity in RAW264.7 macrophages [7]. Zhang et al. [8] further demonstrated that *Polygonatum* polysaccharides activate the NF-κB and p38 MAPK signaling pathways, enhancing macrophage phagocytic function. In addition, a study using an immunosuppressed chicken model revealed that *Polygonatum* polysaccharides could inhibit apoptosis in immune organs such as the spleen and thymus, while upregulating the expression of immune-related genes including *IL-2*, *IL-6*, and *IFN-γ* [9].

Zebrafish (*Danio rerio*) possess an immune system that is evolutionarily conserved and functionally analogous to that of humans, in terms of both the composition of immune cells and functional molecular mechanisms [10]. Their immune system includes neutrophils, macrophages, B cells, and T cells, which represent both innate and adaptive immunity [11]. Furthermore, zebrafish share highly homologous immune-related genes with humans, which establishes them as an ideal model organism for studying human diseases [12]. To establish the immunodeficient model, the *Tg (mpx:GFP)* transgenic zebrafish line could be used, in which neutrophils are specifically labeled with green fluorescent protein [13]. Vinorelbine tartrate is recognized for its ability to induce immunodeficiency by inhibiting bone marrow hematopoiesis [13,14]. This suppression results in decreased levels of platelets, erythrocytes, and various leukocyte subsets, particularly neutrophils, thereby impairing immune function [13,14]. Up to now, there have been limited research reports on the evaluation of the immune-promoting activity of PCP using a zebrafish model. It is essential to conduct in-depth research on utilizing zebrafish as an animal model for evaluating the immunomodulatory effects of *Polygonatum* polysaccharides.

In this study, we employed vinorelbine tartrate to establish an immunodeficiency zebrafish model to evaluate the immunomodulatory activity of PCP. Neutrophil counts and the expression levels of immune-related genes were used as primary indicators. To gain molecular-level insights into the immunomodulatory effects of PCP, transcriptomic analysis was performed to identify differentially expressed genes and signaling pathways potentially involved in the response to PCP treatment. The expression patterns of key candidate genes identified from the transcriptome data were subsequently validated using qRT-PCR. Our findings aim to provide a scientific basis for further exploring the immunological effects of PCP.

## 2. Materials and Methods

### 2.1. Materials

*Polygonatum cyrtonema* Hua was provided by Anhui Dabie Mountain Traditional Chinese Medicine Decoction Co., Ltd. (Lu’an, China). Monosaccharide standards (mannose, rhamnose, glucuronic acid, galacturonic acid, glucose, galactose, arabinose, and fructose) and Dextran standards were purchased from Aladdin Bio-Chem Technology Co., Ltd. (Shanghai, China). The TRIzol Reagent for RNA extraction was obtained from Invitrogen (Shanghai, China), while the PrimeScript RT reagent Kit and SYBR Premix Ex Taq II were purchased from TaKaRa (Beijing, China). Vinorelbine tartrate injection was supplied by Jiangsu Hansoh Pharmaceutical Co., Ltd. (Lianyungang, China). Bailing capsules, a Chinese patent medicine used as a positive control for immunity enhancement [15,16,17], were provided by Hangzhou Zhongmei Huadong Pharmaceutical Co., Ltd. (Hangzhou, China). All other chemicals were of analytical grade.

### 2.2. Experimental Zebrafish

Transgenic neutrophil fluorescent zebrafish *Tg (mpx:GFP)* larvae (3 dpf), in which sex is not yet distinguishable, were maintained at Hunter Biotechnology (Hangzhou, China) under the experimental animal use license SYXK (Zhe) 2022-0004. Their care and management strictly complied with international AAALAC certification (certification number: 001458) and were approved by IACUC ethical review (review number: IACUC-2024-8669-01). The zebrafish were housed in a controlled environment with a water temperature of 28 °C. Water quality was maintained as follows: for every 1 L of reverse-osmosis water, 200 mg of instant sea salt was added to maintain conductivity between 450 and 550 μS·cm^−1^. The pH was kept within 6.5–8.5, and water hardness was maintained at 50–100 mg·L^−1^ CaCO_3_.

### 2.3. Polysaccharides from Polygonatum cyrtonema Hua

#### 2.3.1. Preparation of *Polygonatum cyrtonema* Polysaccharides

Polysaccharides from *Polygonatum cyrtonema* Hua were extracted using the water extraction and alcohol precipitation method. The filtrate was collected, concentrated, and subjected to alcohol precipitation with anhydrous ethanol. The supernatant was discarded, and crude polysaccharides were obtained. Protein removal was carried out using the Sevag reagent. The aqueous polysaccharide solution was then freeze-dried to obtain *Polygonatum cyrtonema* polysaccharide powder. The total sugar content was determined by using the anthrone–sulfuric acid method, and the protein content was quantified using a modified BCA protein assay kit (Sangon Biotech, Shanghai, China).

#### 2.3.2. FTIR Spectroscopy Analysis

FTIR spectroscopy (Thermo Scientific, Shanghai, China) was employed to characterize the organic functional groups in the polysaccharides. The spectra were recorded in the range of 4000–500 cm^−1^ with a resolution of 4 cm^−1^. The dried polysaccharides sample was mixed with potassium bromide at a ratio of 1:100 and pressed into pellets for measurement. Peak assignments were performed using OMNIC 8.2 software.

#### 2.3.3. Molecular Weight Distribution Analysis

The molecular weight distribution of PCP was determined by high-performance gel permeation chromatography (HPGPC). Dextran standards with known molecular weights (5, 10, 20, 40, 70, 150, 280, 1000, and 2000 kDa) and PCP were each dissolved in ultrapure water to a final concentration of 2 mg·mL^−1^. The solutions were filtered through a 0.45 μm membrane prior to analysis. Chromatographic separation was performed on a TSK-GEL GMPWXL column (7.8 × 300 mm, 10 μm) equipped with a refractive index detector. The column temperature was maintained at 25 °C. Ultrapure water was used as the mobile phase at a flow rate of 0.5 mL·min^−1^, and the injection volume was 20 μL. A calibration curve was constructed using the retention time of each dextran standard as the independent variable and the logarithm of molecular weight (log Mw) as the dependent variable. The relative molecular weight of PCP was calculated by interpolating the retention times of the sample peaks into the calibration curve.

#### 2.3.4. Monosaccharide Composition Analysis

Monosaccharide composition was analyzed according to the method of Ping Zhao et al. [18]. Briefly, a 10 mg sample was hydrolyzed with 2 mol·L^−1^ trifluoroacetic acid at 110 °C for 2 h, followed by pre-column derivatization with 1-phenyl-3-methyl-5-pyrazolone (PMP) at 70 °C for 2 h. The monosaccharides (except fructose) were separated on an Agilent 1290 HPLC system equipped with an Agilent Extend-C18 column (4.6 × 250 mm, 5 μm). The mobile phase consisted of solvent A (0.02 mol·L^−1^ ammonium acetate) and solvent B (acetonitrile), using the following gradient elution program: 0–5 min, solvent A from 60% to 90%; 5–12 min, 90% to 85%; 12–20 min, 85% to 80%; 20–30 min, 80% to 75%; 30–40 min, 75% to 70%; 40–45 min, 70% to 60%; 45–50 min, 60% to 55%; 50–52 min, 55% to 0%; 52–55 min, held at 0%; 55–56 min, 0% to 97%; 56–57 min, held at 97%. The flow rate was maintained at 0.25 mL·min^−1^, the column temperature was controlled at 30 °C, and UV detection was conducted at a wavelength of 250 nm.

Fructose, which is not amenable to PMP derivatization, was analyzed separately using a high-performance liquid chromatography (HPLC) method. A 10 mg sample was hydrolyzed with 0.05 mol/L TFA for 30 min, at 110 °C in sealed glass ampules. The analysis was carried out on an Agilent 1290 HPLC system fitted with a Hypersil NH_2_ column (4.6 mm × 250 mm, 5.0 μm) at 35 °C. The mobile phase consisted of acetonitrile and ultrapure water (85:15), and the flow rate was set at 1.0 mL·min^−1^. Detection was achieved using an evaporative light scattering detector (ELSD) with nebulizer and evaporator temperatures set at 60 °C and 40 °C, respectively. Prior to analysis, all samples were filtered through a 0.45 μm organic membrane. Quantification of fructose was performed by comparison of retention times and peak areas with those of fructose standards.

In the quantitative analysis of monosaccharide composition, the molar percentages of each monosaccharide component were determined according to the method described by Ping Zhao et al. [18]. The calculation formula is as follows:

$$m_{x}\%=\frac{\left(A_{x}\times C_{i}\times V_{x}/A_{i}\right)}{\sum_{x=1}^{n}\left(A_{x}\times C_{i}\times V_{x}/A_{i}\right)}\times 100\%$$

where *A_x_* is the peak area of monosaccharide in the sample; *A_i_* is the peak area of the standard; *C_i_* is the molar concentration of the standard; and *V_x_* is the volume of the sample solution.

### 2.4. Effects of Polygonatum cyrtonema Polysaccharides in a Zebrafish Immunodeficiency Model

Transgenic zebrafish larvae expressing green fluorescent neutrophils (*Tg (mpx:GFP)*) at 3 days postfertilization (dpf) were used to establish an immunodeficiency model. Except for the normal control group, all larvae were intravenously microinjected with vinorelbine tartrate (200 μg·mL^−1^, 10 nL) to induce immunodeficiency. The criterion for confirming the successful development of the immunodeficient zebrafish model was a statistically significant reduction in neutrophil numbers compared to the normal control group (*p* < 0.05) [13]. Each experimental well contained 3 mL of solution, and the study included both a normal control group and a model control group. To evaluate the maximum tolerated concentration of PCP, 30 zebrafish larvae in each group were exposed to aqueous PCP solutions at 2000, 1000, 500, 250, 125, 62.5, and 31.2 μg·mL^−1^. Following 48 h incubation at 28 °C, the MTC was determined based on larval survival and overall physiological condition.

For efficacy studies, 30 zebrafish larvae per well were exposed to 500, 1000, 2000 μg·mL^−1^ PCP and 15 μg·mL^−1^ Bailing capsules. After 48 h of incubation at 28 °C, 10 zebrafish were randomly selected from each group and subjected to fluorescence imaging under a fluorescence microscope. Neutrophil counts in the caudal vein were quantified using NIS-Elements D 3.20 advanced imaging software.

### 2.5. Zebrafish mRNA Sequencing

For mRNA sequencing, zebrafish larvae were collected from the same experimental batches used for neutrophil quantification (Section 2.4), including the model group (MX) and PCP-treated groups at 500 (P500), 1000 (P1000), and 2000 μg/mL (P2000). Whole-body larvae were used for RNA extraction. For each group, 10 larvae were pooled as one biological replicate, and three independent biological replicates were prepared. Prior to RNA extraction, larvae were rinsed three times with sterile PBS and immediately frozen in liquid nitrogen. Total RNA from zebrafish was extracted using TRIzol (Invitrogen, Shanghai, China). RNA concentration and purity were assessed using a NanoDrop 2000 spectrophotometer (Thermo Fisher Scientific, Shanghai, China), with acceptable quality defined as OD260/OD280 ≥ 1.8 and OD260/OD230 ≥ 1.5. Transcriptome sequencing was performed on the BGISEQ platform, which was based on the combinatorial probe-anchor synthesis (cPAS) technology and enhanced DNA nanoball (DNB) sequencing method. Library preparation and RNA-Seq were conducted by BGI Genomics Co., Ltd. (Shenzhen, China). A dedicated library construction kit was used to prepare sequencing libraries according to the manufacturer’s standard protocol.

### 2.6. Bioinformatics Analysis

Raw sequencing reads were processed using the Trimmomatic (version 0.39) package on a CentOS server to improve sequence quality and ensure reliability for downstream analyses. Clean reads were then aligned to the zebrafish reference genome (GRCz11-GCF_000002035.6) using HISAT2 (version 2.2.1). Alignment results were processed with StringTie (version 2.2.1) to generate transcript annotation files, which were subsequently merged into a comprehensive annotation file using StringTie. Transcript and gene expression quantification were performed using the Cuffdiff program (version 2.2.1), which generated a diff_out directory. Differential expression analysis results were further analyzed using the cummeRbund package in R (version 2.40.0) to explore gene expression distributions, identify DEGs, and visualize expression profiles. To further investigate the biological significance of DEGs in terms of biological processes, cellular components, and molecular functions, GO and KEGG enrichment analyses were conducted. A custom-built script was developed to generate background databases compatible with the ClusterProfiler R package (version 4.10.0) for GO and KEGG enrichment analyses. DEGs between experimental groups were subjected to enrichment analysis, from which significantly enriched GO terms and KEGG pathways were identified. Finally, the integration of enrichment results enabled the identification of metabolic and signaling pathways differentially regulated among sample groups, providing insights into the underlying biological mechanisms.

### 2.7. Real-Time Quantitative PCR Validation

Several DEGs (*il1b*, *crk*, *fgf10b*, *atp6v1aa*, *eif4e1c*) were validated via RT-qPCR. Total RNA was reverse-transcribed using TaKaRa kits, and amplification was performed using SYBR Premix Ex Taq II on a LightCycler 96 (Roche, Basel, Switzerland). The reverse transcription reaction was performed using the following protocol: incubation at 37 °C for 15 min, followed by inactivation at 85 °C for 5 s, and final storage at 4 °C for subsequent use. The PCR thermocycling protocol was conducted as follows: initial denaturation at 95 °C for 30 s (1 cycle), followed by 40 cycles of denaturation at 95 °C for 5 s and annealing/extension at 60 °C for 30 s, then a final extension at 95 °C for 1 min, followed by cooling at 50 °C for 30 s (1 cycle). Melting curve analysis was performed after amplification to confirm the specificity of each PCR product. Relative gene expression levels were calculated using the 2^−ΔΔCt^ method [19], with *β-actin* serving as an internal reference and the MX group used as the calibrator for each target gene. The primers utilized in the RT-qPCR analysis are listed in Table 1.

### 2.8. Statistical Analyses

Data are expressed as the mean ± standard error of the mean (SEM). Statistical analyses were performed using GraphPad Prism 8.0.2 (GraphPad Software, San Diego, CA, USA, www.graphpad.com, accessed on 15 July 2025) through a one-way ANOVA followed by Dunnett’s *t*-test.

## 3. Results

### 3.1. Composition of Polysaccharides Extracted from Polygonatum cyrtonema Hua

#### 3.1.1. Chemical Composition and FTIR Analysis of *Polygonatum cyrtonema* Polysaccharides

**Figure 1:**
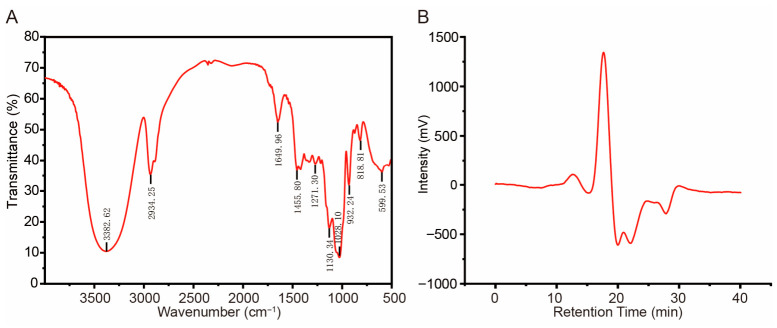
Structural characteristics and molecular weight distribution of the *Polygonatum cyrtonema* Polysaccharides (PCP). (**A**) Fourier-transform infrared spectroscopy (FTIR) spectrum of the polysaccharides. (**B**) High-performance gel permeation chromatography (HPGPC) profile showing the molecular weight distribution of the polysaccharides.

The crude PCP was obtained from the raw material using hot water extraction and ethanol precipitation, followed by purification with the Sevag reagent. The total sugar content of PCP was determined to be 80.8 ± 1.9%, while the residual protein content was 0.93 ± 0.05%. FTIR spectroscopy analysis (Figure 1A) revealed characteristic polysaccharide functional group absorption peaks. A broad absorption peak at 3382.62 cm^−1^ corresponds to O-H stretching vibrations, indicating abundant hydroxyl groups. The peak at 2934.25 cm^−1^ represents C-H stretching vibrations, suggesting the presence of saturated hydrocarbon groups. The absorption peak at 1649.96 cm^−1^ is attributed to C=O stretching, while the peak at 1455.80 cm^−1^ corresponds to CH_3_ ending vibrations. In the fingerprint region (1300–500 cm^−1^), the peak at 1271.30 cm^−1^ is associated with C-O glycosidic bond stretching. Peaks at 1130.34 cm^−1^ and 1028.10 cm^−1^ result from asymmetric C-O-C stretching vibrations in the pyran ring. The absorption peak at 932.24 cm^−1^ corresponds to β-glycosidic bond vibrations of fructose, while the peak at 818.81 cm^−1^ indicates the presence of α-glycosidic bonds. The results suggest that PCP had the characteristic absorption of typical polysaccharides.

#### 3.1.2. Molecular Weight Distribution

Based on dextran standards, the standard curve for molecular weight determination was established as *Y* = −0.3368*X* + 9.4331, showing a strong linear correlation (R^2^ = 0.9902), confirming the reliability of the HPGPC system. The molecular weight distribution profile of PCP is shown in Figure 1B. A minor peak was observed at a retention time of 12.58 min; however, the peaks eluting between 10.99 and 12.74 min exceeded the effective detection range of the refractive index detector, with estimated molecular weights ranging from approximately 138.76 to 539.35 kDa. In contrast, a dominant and well-defined peak appeared at 17.28 min. Based on retention times between 16.05 and 18.16 min and interpolation from the standard curve, the molecular weight of the refined PCP fraction was determined to be in the range of 2.07 to 10.65 kDa. This indicates that low-molecular-weight polysaccharides constituted the main components of the purified sample.

#### 3.1.3. Monosaccharide Composition of PCP

**Figure 2:**
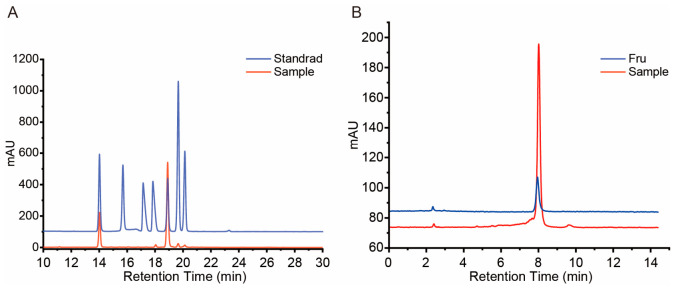
HPLC analysis of the monosaccharide composition of *Polygonatum cyrtonema* polysaccharides (PCP). (**A**) PMP-derivatized HPLC chromatograms showing the separation of mixed monosaccharide standards (blue line) and acid-hydrolyzed PCP sample (orange line). The distinct peaks in the sample profile indicate the presence of different monosaccharide residues in PCP. (**B**) HPLC chromatograms for quantitative determination of fructose in PCP. Blue line: fructose standard; orange line: PCP hydrolysate.

HPLC analysis of PCP was performed following complementary hydrolysis and derivatization methods based on Zhao et al. [18]. As shown in Figure 2A, the HPLC profile of PCP (orange line) displayed well-resolved peaks that co-eluted with monosaccharide standards (blue line), confirming the presence of mannose (Man), rhamnose (Rha), glucuronic acid (GlcA), galacturonic acid (GalA), glucose (Glc), galactose (Gal), and arabinose (Ara) in the polysaccharide fraction. Figure 2B presents the chromatogram for fructose analysis, in which the dominant peak in the PCP hydrolysate (orange line) precisely co-eluted with the fructose (Fru) standard (blue line), indicating a high fructose content. Quantitative analysis showed that fructose was the dominant monosaccharide, constituting 70.00% of the total. This was followed by glucose (22.36%) and mannose (6.39%). Minor components included galacturonic acid and arabinose (each 0.46%) and galactose (0.33%). Accordingly, the molar ratio of Man, GalA, Glc, Gal, Ara and Fru in PCP was calculated as 6.39:0.46:22.36:0.33:0.46:70.0. The exceptionally high fructose proportion suggests that fructose residues are the principal structural units of PCP. According to the findings of Ping Zhao et al. [18], fructose in *Polygonatum* polysaccharides is primarily polymerized in the form of fructans rather than existing as free monosaccharides. This structural feature supports the classification of PCP as a fructan-rich polysaccharide, in agreement with previous reports on *Polygonatum* species [18,20]. Importantly, fructans have been widely reported to possess immunomodulatory activities, including the enhancement of innate and adaptive immune responses [21,22,23,24,25,26]. Therefore, the fructan-dominant composition of PCP provides a plausible structural basis for its observed immune enhancement effects.

### 3.2. Effects of Polysaccharides of Different Concentrations in a Zebrafish Immunodeficiency Model

To evaluate the immune-enhancing effects of PCP at different concentrations in vivo, a zebrafish immunodeficiency model was established. In this model, neutrophils were labeled with green fluorescence for visualization, and immunodeficiency was induced by microinjecting vinorelbine tartrate into the caudal vein. Zebrafish were exposed to PCP at 31.2, 62.5, 125, 250, 500, 1000, and 2000 μg·mL^−1^ to assess both toxicity and immunomodulatory activity. Across all PCP concentrations tested, no mortality (0/30 larvae in each group) and no detectable toxic phenotypes were observed in immunodeficient zebrafish. The physiological condition and morphology of zebrafish in all treatment groups were comparable to those of the model control group. As no adverse effects occurred at the highest concentration tested (2000 μg·mL^−1^), the maximum tolerated concentration (MTC) of PCP was determined to be greater than 2000 μg/mL under the present experimental conditions (Supplementary Table S1). Based on these results, 500, 1000, and 2000 μg·mL^−1^ were selected as the low-, medium-, and high-dose groups for subsequent analyses.

**Figure 3:**
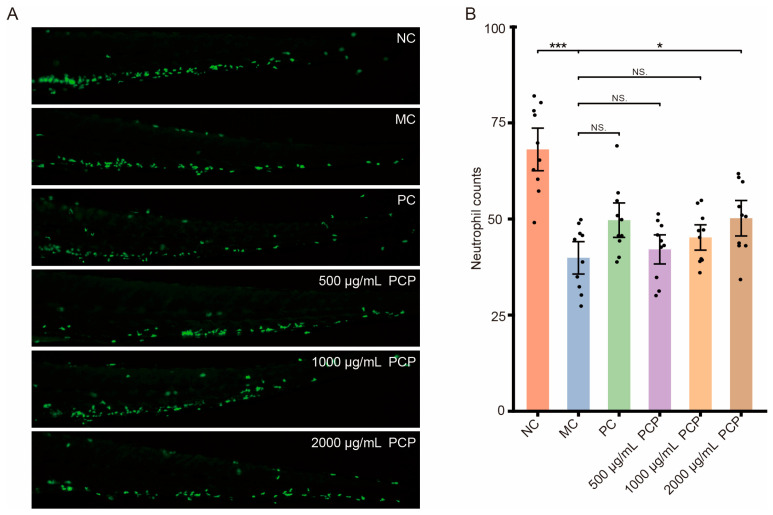
*Polygonatum cyrtonema* polysaccharide (PCP) treatment improves vinorelbine-induced neutropenia and promotes neutrophil recovery in immunodeficient zebrafish. (**A**) Representative in vivo fluorescence images showing neutrophil distribution in *Tg (mpx:GFP)* zebrafish. NC: normal control group; MC: vinorelbine-induced model control group; PC: positive control group (15 μg·mL^−1^ Bailing capsule); 500, 1000, and 2000 μg·mL^−1^ PCP: zebrafish treated with gradient concentrations of *Polygonatum cyrtonema* polysaccharides. (**B**) Quantitative statistical analysis of caudal vein neutrophil abundance in different groups. Each dot represents an individual zebrafish sample. Data are presented as mean ± SEM, *n* = 10. Statistical analysis was performed using one-way ANOVA with Dunnett’s post hoc test. * *p* < 0.05, *** *p* < 0.001. NS: no significant difference, compared with the MC group.

As depicted in Figure 3 (MC label), compared with the normal control group (NC label), the number of neutrophils in the caudal region of zebrafish markedly decreased following vinorelbine tartrate injection. As shown in Table 2, this reduction was statistically significant (*p* < 0.001), confirming that the zebrafish immunodeficiency model was successfully established. Figure 3 (PC, 500 μg·mL^−1^ PCP, 1000 μg·mL^−1^ PCP, and 2000 μg·mL^−1^ PCP label) demonstrated that treatment with the positive control drug (Bailing capsules) and PCP at various concentrations exerted immune-enhancing effects to varying degrees in immunodeficient zebrafish. Compared to the model group, neutrophil counts in zebrafish treated with 15 μg·mL^−1^ Bailing capsules and 500, 1000, and 2000 μg·mL^−1^ PCP were 49.7 ± 2.84, 42.1 ± 2.40, 45.2 ± 2.09, 50.2 ± 2.92 cells per fish, respectively (Table 2). Notably, 2000 μg·mL^−1^ PCP significantly increased neutrophil numbers compared to the model group (*p* < 0.05, Table 2), indicating immune-enhancing activity.

### 3.3. Mapping and Analysis of Zebrafish RNA-Seq Data

To investigate the transcriptomic changes in zebrafish induced by PCP, RNA-seq was performed to compare the treated group with the control. Sequencing of 12 samples on the BGISEQ platform produced an average of 6.71 Gb of data per sample. After quality control, the reads were aligned to the zebrafish reference genome (GRCz11-GCF_000002035.6), yielding an average genome alignment rate of 96.33% and an average gene alignment rate of 72.27%, which confirmed the high data quality for subsequent transcriptomic analysis.

As shown in Table 3, the sequencing data exhibited high quality across all samples. The proportion of clean reads with Q20 scores exceeded 97%, indicating a low base-calling error rate. The Q30 scores ranged from 91.76% to 92.92%, further confirming data fidelity. Additionally, 92% to 96% of raw reads were retained as clean reads, demonstrating efficient filtering. The above results confirm that the RNA-seq data are robust and suitable for downstream transcriptomic analysis.

### 3.4. Analysis of Differentially Expressed Genes in Zebrafish

**Figure 4:**
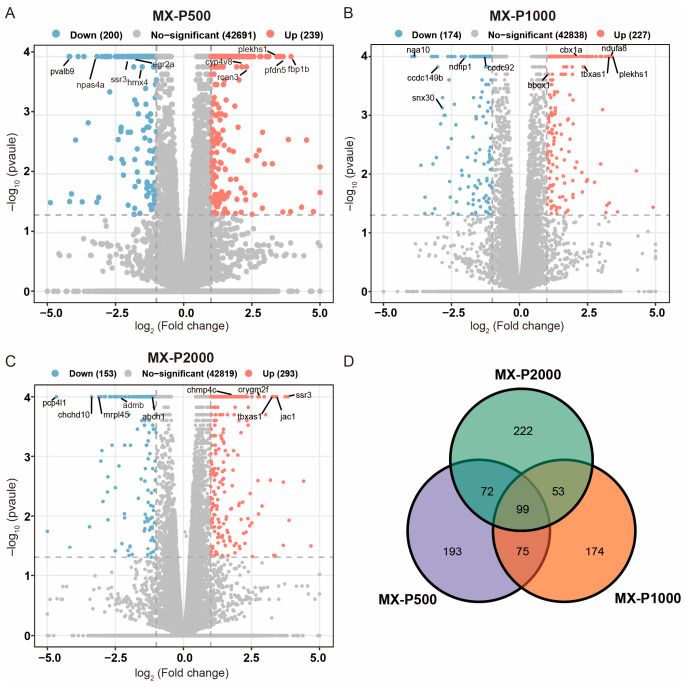
Transcriptomic profiling of differentially expressed genes (DEGs) in zebrafish following *Polygonatum cyrtonema* polysaccharide (PCP) treatment. (**A**–**C**) Volcano plots of DEGs in model control (MX)- versus PCP-treated groups: 500 μg·mL^−1^ (P500), 1000 μg·mL^−1^ (P1000), and 2000 μg·mL^−1^ (P2000). The x-axis represents the log_2_ fold change (FC), and the y-axis represents the −log_10_ (*p*-value). Upregulated genes (log_2_ FC ≥ 1, *p* < 0.05) are shown in orange, and downregulated genes (log_2_ FC ≤ −1, *p* < 0.05) are shown in blue. Statistically non-significant genes (gray) fall outside these thresholds. Selected genes with pronounced differential expression are labeled. (**D**) Venn diagram showing the overlap of DEGs among the three PCP treatment comparisons (MX-P500, MX-P1000, and MX-P2000). The numbers in the Venn diagram represent the counts of unique and common DEGs across the compared groups.

To examine transcriptomic alterations in zebrafish induced by PCP, DEGs were identified by comparing each treatment group to the control using the thresholds of |log_2_ fold change| ≥ 1 and *p* < 0.05. The results are visualized in volcano plots and a Venn diagram (Figure 4). In the volcano plots, upregulated genes (log_2_ fold change ≥ 1) are shown in orange on the right, and downregulated genes (log_2_ fold change ≤ −1) in blue on the left.

PCP treatment induced substantial transcriptomic changes in a dose-dependent manner. The low-dose group (500 μg·mL^−1^, MX-P500) showed 439 identified DEGs, including 239 upregulated and 200 downregulated genes (Figure 4A). The medium-dose group (1000 μg·mL^−1^, MX-P1000) had 401 DEGs (227 up, 174 down; Figure 4B). The high-dose group (2000 μg·mL^−1^, MX-P2000) exhibited the greatest number of DEGs (446 total; 293 up, 153 down; Figure 4C).

Venn diagram analysis revealed overlapping DEGs across groups (Figure 4D). A total of 174 DEGs were shared between MX-P500 and MX-1000, 171 DEGs were shared between MX-P500 and MX-P2000, and 152 DEGs were shared between MX-P1000 and MX-P2000. Notably, 99 DEGs were common to all three groups, suggesting a core set of genes involved in the zebrafish response to PCP.

### 3.5. Functional Annotation and Analysis of Differentially Expressed Genes

To elucidate the functional roles of DEGs in zebrafish following treatment with low (500 μg·mL^−1^), medium (1000 μg·mL^−1^), and high (2000 μg·mL^−1^) concentrations of PCP, GO and KEGG enrichment analyses were performed. Only pathways with a *p* value < 0.05 were considered significantly enriched, with a particular focus on immune-related processes.

#### 3.5.1. GO Enrichment Analysis

**Figure 5:**
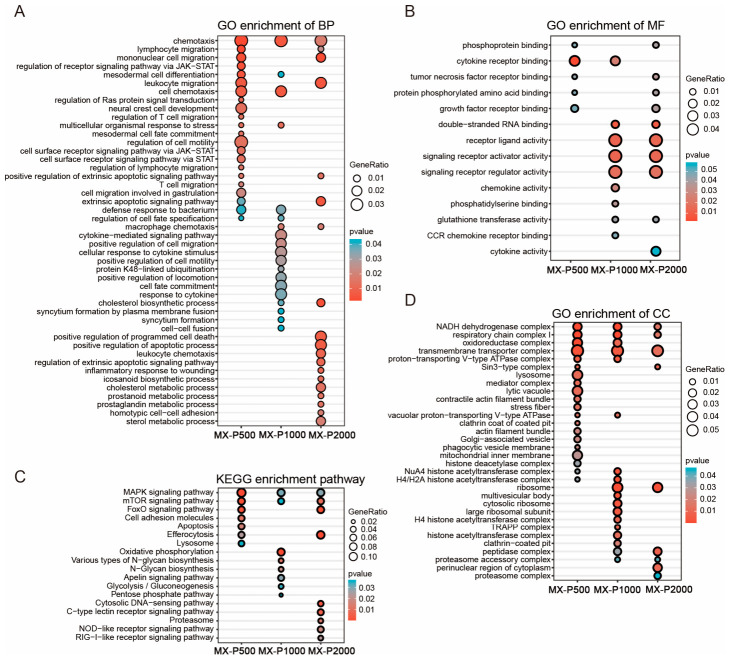
Functional enrichment analysis of differentially expressed genes (DEGs) following PCP treatment in zebrafish. (**A**) GO Biological Process (BP) enrichment analysis. (**B**) GO Molecular Function (MF) enrichment analysis. (**C**) Kyoto Encyclopedia of Genes and Genomes (KEGG) pathway enrichment analysis. (**D**) GO Cellular Component (CC) enrichment analysis. Each bubble represents an enriched functional pathway. The size of the bubble indicates the gene ratio, and the color gradient indicates the *p*-value. Terms with *p*-value < 0.05 were considered significantly enriched.

GO enrichment analysis categorized the DEGs into the biological process (BP), cellular component (CC), and molecular function (MF) domains. Enrichment in the BP category (Figure 5A) was primarily linked to immune cell activation and developmental processes, such as chemotaxis (GO:0006935), lymphocyte migration (GO:0072676), mononuclear cell migration (GO:0071674), leukocyte migration (GO:0050900), and macrophage chemotaxis (GO:0048246). Other enriched terms included mesodermal cell differentiation, multicellular organismal response to stress, and several apoptotic signaling pathways, indicating potential involvement of PCP in immune regulation and tissue remodeling.

In the MF category (Figure 5B), significantly enriched terms were associated with immune-related receptor interactions and signaling activities, including cytokine receptor binding (GO:0005126), tumor necrosis factor receptor binding (GO:0005164), receptor ligand activity (GO:0048018), signaling receptor activator activity (GO:0030546), and double-stranded RNA binding (GO:0003725). These functions suggest activation of immune signaling cascades and modulation of host defense mechanisms.

As shown in the cellular component (CC) analysis (Figure 5D), the identified DEGs were primarily enriched in protein complexes involved in cellular energy metabolism, protein turnover, and transcriptional regulation. Key enriched terms comprised the NADH dehydrogenase complex (GO:0030964), respiratory chain complex I (GO:0045271), ribosome (GO:0005840), proteasome accessory complex (GO:0022624), and several histone acetyltransferase complexes. These findings collectively suggest that PCP treatment likely perturbs mitochondrial function and may concurrently influence epigenetic regulatory pathways.

Collectively, these findings indicate that the immunomodulatory activity of PCP in zebrafish is mediated through two primary mechanisms: facilitating the migration and chemotaxis of key immune cells (lymphocytes, monocytes, and macrophages) and orchestrating immune receptor signaling alongside cellular energy metabolism. Notably, this activity was dose-dependent, with more pronounced functional enrichment observed at higher PCP concentrations. This highlights the potential of PCP as a bioactive immunomodulator capable of enhancing host immune responses.

#### 3.5.2. KEGG Pathway Enrichment Analysis

To elucidate the molecular mechanisms by which PCP modulates immune function, KEGG pathway enrichment analysis was performed on DEGs from immunosuppressed zebrafish (model control group, MX) treated with low (P500, 500 μg·mL^−1^), medium (P1000, 1000 μg·mL^−1^), and high (P2000, 2000 μg·mL^−1^) doses of PCP (Figure 5C). The analysis identified significantly enriched pathways (*p* < 0.05), particularly those associated with immune regulation, signal transduction, and metabolism, indicating that PCP exerts comprehensive modulatory effects on the immune system.

The MAPK (dre04010) and mTOR (dre04150) signaling pathways were enriched across all dose groups, highlighting their central role in PCP-mediated immune activation and cellular growth. The FoxO signaling pathway (dre04068), a regulator of immune homeostasis and oxidative stress [27], was significantly enriched in the P500 and P2000 treatment groups. High-dose PCP (P2000) upregulated several pathways associated with innate immunity, including the Cytosolic DNA-sensing (dre04623), C-type lectin receptor (dre04625), NOD-like receptor (dre04621), and RIG-I-like receptor (dre04622) signaling pathways, indicating a potent engagement of pattern recognition receptors. In the P1000 group, enrichment of oxidative phosphorylation (dre00190), glycolysis/gluconeogenesis (dre00010), and the pentose phosphate pathway (dre00030) indicated a metabolic reprogram which supports the energy demands of immune recovery. Additionally, enhanced activity of efferocytosis and lysosome pathways in the P2000 group reflected improved clearance of apoptotic cells and antigen processing capacity under high PCP exposure.

In summary, PCP could modulate key immune and metabolic pathways in immunodeficient zebrafish. The MAPK and mTOR pathways form a core response across doses; high-dose PCP additionally activates innate immune sensing and cell clearance mechanisms. These findings reveal the multifaceted mechanism behind PCP’s immunostimulatory activity and provide a rationale for its application in immune enhancement.

### 3.6. Validation of Immune-Related Differentially Expressed Genes

**Figure 6:**
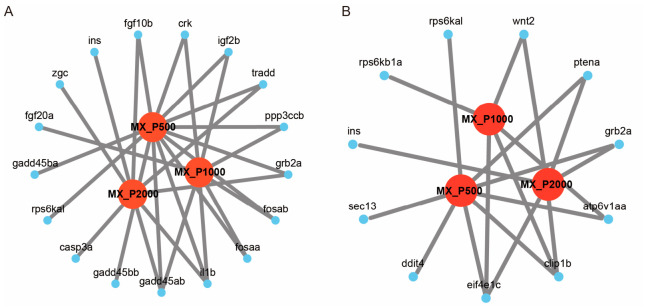
Visualization of genes involved in immune-related KEGG pathways. (**A**) The pattern-recognition-related “MAPK signaling pathway”. (**B**) The pattern-recognition-related “mTOR signaling pathway”. Red nodes represent the PCP treatment groups; blue nodes represent pathway-related differentially expressed genes (DEGs).

To validate the transcriptomic data and further investigate the immunomodulatory effects of PCP, several differentially expressed genes (DEGs) including *il1b*, *crk*, *fgf10b*, *atp6v1aa*, and *eif4e1c* were selected from significantly enriched KEGG pathways (the MAPK signaling pathway and the mTOR signaling pathway) for RT-qPCR analysis, based on their biological relevance and expression significance across treatment groups. The gene expression data for the MAPK and mTOR signaling pathways, including gene ID, gene symbol, and log_2_fc values from the RNA-Seq analysis, are provided in Supplementary Table S2 and Figure 6. In the MAPK signaling pathway, a total of 13 DEGs were identified in the MX-P500 group according to the RNA-Seq data; among these, *ppp3ccb*, *il1b*, *crk*, and *fgf10b* were upregulated, whereas nine other *genes*, including *gadd45ab* and *tradd*, were downregulated. In the MX-P1000 group, seven DEGs were detected, with *fgf20a* and *crk* showing marked upregulation and the remaining five genes being downregulated. In the MX-P2000 group, eight DEGs were identified, among which *il1b*, *ins*, *zgc*, and *fgf10b* were upregulated, and the other four genes were downregulated. The gene *il1b* encodes a classic pro-inflammatory cytokine and is indicative of immune activation [28]. *Gadd45ab*, involved in DNA damage repair and immune tolerance, was consistently downregulated and may reflect a release of immunosuppressive stress [29]. *Crk* is a known adaptor protein in immune signaling cascades [30], while *fgf10b* is associated with cell proliferation and tissue regeneration [31]. *Tradd*, a key adaptor in TNF receptor-mediated signaling and apoptosis, serves as a marker of immune suppression and was downregulated in two treatment groups [32].

**Figure 7:**
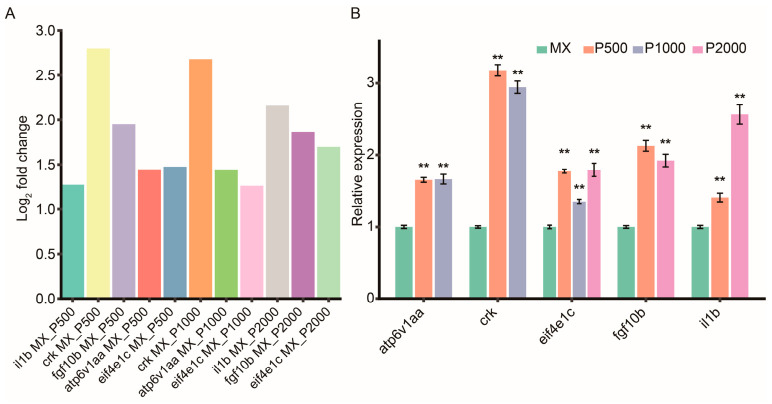
Validation of selected differentially expressed genes (DEGs) by RT-qPCR. (**A**) Log_2_ fold change in candidate differentially expressed genes (DEGs) identified by RNA-Seq in zebrafish treated with *Polygonatum cyrtonema* polysaccharides (PCP) at concentrations of 500 (P500), 1000 (P1000), and 2000 (P2000) μg·mL^−1^, relative to the Model Control (MX) group. (**B**) Relative mRNA expression levels of the corresponding genes determined by RT-qPCR using the 2^−ΔΔCt^ method. Data are presented as mean ± SD (*n* = 3). Statistical significance was analyzed by one-way ANOVA followed by Tukey’s multiple comparison test. Asterisks denote ** *p* < 0.01 compared with the MX group. Abbreviations: *il1b*, interleukin 1 beta; *crk*, v-crk avian sarcoma virus CT10 oncogene homolog; *fgf10b*, fibroblast growth factor 10b; *atp6v1aa*, ATPase H+ transporting V1 subunit Aa; *eif4e1c*, eukaryotic translation initiation factor 4E family member 1c.

In the mTOR signaling pathway, eight DEGs were detected in the MX-P500 group, with upregulation of *clip1b*, *eif4e1c*, and *atp6v1aa* and downregulation of five genes including *ptena*. In the MX-P1000 group, five DEGs were identified, including upregulation of *clip1b*, *eif4e1c*, and *atp6v1aa*. Six DEGs were observed in the MX-P2000 group, with *eif4e1c*, *clip1b*, and *ins* being significantly upregulated. Among these, *eif4e1c* plays a critical role in translation initiation and T cell activation [33]; *clip1b* is involved in cytoskeletal regulation downstream of mTOR activation [34]; *atp6v1aa* contributes to immune lysosomal activity, potentially indicating enhanced immune cell function [35]; and *ptena*, a well-known negative regulator of the PI3K-Akt-mTOR pathway [36], was consistently downregulated, further supporting pathway activation. Based on these results, representative immune-related DEGs were selected for RT-qPCR validation, including *il1b*, *crk*, *fgf10b*, *eif4e1c*, and *atp6v1aa*, with selection tailored across the three treatment groups. As shown in Figure 7, the RT-qPCR results displayed expression trends consistent with the log_2_fc value of RNA-Seq data, confirming the reliability of the transcriptomic analysis. The expression levels of candidate genes were higher in several treatment groups—*crk*, *fgf10b*, *atp6v1aa*, and *eif4e1c* in MX-P500; *crk* and *atp6v1aa* in MX-P1000; and *il1b*, *fgf10b*, and *eif4e1c* in MX-P2000—compared to *il1b* in MX-P500 and *eif4e1c* in MX-P1000. These findings validate the activation of the MAPK and mTOR signaling pathways and demonstrate that PCP exerts its immunostimulatory effects by enhancing pro-inflammatory signaling, cellular proliferation, and translational activation.

## 4. Discussion

PCPs have been reported to exert immunomodulatory effects in different models, including macrophage activation and immunosuppressed animals [3,24,37,38]. In contrast, LPS, derived from Gram-negative bacteria and consisting of lipid A, core oligosaccharide and O-antigen, triggers excessive inflammatory responses by activating the NF-κB and MAPK pathways through the TLR4/MD2 complex [39,40]. Although LPS and plant-derived polysaccharides can interact with pattern recognition receptors such as TLR4 [41], they induce different immunological responses. Plant polysaccharides primarily maintain immune homeostasis by moderately activating downstream signaling cascades without triggering excessive inflammation [42]. Their biological activities are closely associated with structural features, including molecular weight, monosaccharide composition, and glycosidic linkages [42]. Previous studies have shown that *Polygonatum* polysaccharides can regulate neutrophil responses and inflammatory signaling via the TLR4–MAPK/NF-κB pathway [41,43]. In our study, PCP showed a high fructose content (70%), confirming its fructan-enriched structural characteristic. In *Polygonatum* polysaccharides, fructose mainly exists in the polymerized fructan form, which confers prominent immunomodulatory potential [24]. Fructans with moderate to high degrees of polymerization are less readily absorbed and are more likely to interact with pattern recognition receptors on innate immune cells [24]. This structural feature provides a potential plausible basis for the ability of PCP to regulate cytokine production and promote neutrophil recovery observed in this study.

Although murine models are commonly used, zebrafish (*Danio rerio*) offer unique advantages in immunopharmacological studies, including high genetic homology with humans, transparent embryos, and rapid development, enabling real-time observation of immune responses and reduced research costs [44,45,46]. Zebrafish are increasingly utilized in immune regulation research, particularly in studies concerning neutrophils, which are the important innate immune cells [47,48,49]. Various zebrafish models of immunodeficiency have been developed, including T cell deficient [50,51], neutropenia [52,53,54], and macrophage deficient models [55]. In particular, the *Tg (mpx:GFP)* transgenic line, which expresses green fluorescent protein specifically in neutrophils, enables intuitive and direct visualization of neutrophil behavior and distribution [47].

In this study, we utilized a genetically modified neutropenic zebrafish *Tg (mpx:GFP)* model to evaluate the immunomodulatory effects of PCP. Our analyses revealed that PCP administration significantly recovered neutrophil numbers in a dose-dependent manner. The high-dose PCP group (MX-P2000) exhibited effects phenotypically comparable to those of the clinical immunomodulator Bailing capsules. This finding is consistent with previous studies using mouse models, as described by Dong Liu et al. [3]. Our zebrafish model investigation demonstrates that PCP exerts a clear immune recovery effect at the phenotypic level. PCP-loaded liposomes have been shown to recover immune organ indices and circulating immune cell populations in immunosuppressed mice [3]. Our results similarly revealed a dose-dependent recovery of neutrophil numbers in vinorelbine-induced immunodeficient zebrafish. Despite differences in formulation strategy and animal models, both studies converge on the conclusion that PCP enhances innate immune cell abundance. This convergence supports the robustness of its immunomodulatory effect across species and enhances its potential translational relevance.

Transcriptomic analysis provided critical insights into the immunomodulatory mechanisms of PCP. GO enrichment analysis of DEGs revealed distinct immune related BP terms activated at each dosage level. In the low-PCP-dose group (MX-P500), enriched GO terms were predominantly associated with immune cell migration and chemotaxis, including lymphocyte migration (GO:0072676), mononuclear cell migration (GO:0071674), leukocyte migration (GO:0050900), cell chemotaxis (GO:0060326), and chemotaxis (GO:0006935). Concurrently, terms such as regulation of receptor signaling via JAK-STAT (GO:0046425) and cell surface receptor signaling via STAT (GO:0097696) were also enriched. This pattern implied that a low dose of PCP may primarily promote the recruitment and positioning of immune cells to sites of challenge, potentially strengthening early immune surveillance and early response signaling. In the medium-dose group (MX-P1000), GO terms extended to encompass macrophage chemotaxis (GO:0048246), cytokine-mediated signaling pathway (GO:0019221), cellular response to cytokine stimulus (GO:0071345), positive regulation of cell migration (GO:0030335), and positive regulation of cell motility (GO:2000147). These findings indicate that innate immune responses were activated, potentially through cytokine signaling and the mobilization of macrophages. The enrichment of terms such as defense responses to bacterium (GO:0042742) and multicellular organismal responses to stress (GO:0033555) further highlight PCP’s capacity to augment the host’s defense system at this stage. With high-dose PCP (MX-P2000), DEG enrichment shifted toward pathways governing inflammation and immune effector functions. Key processes included the positive regulation of programmed cell death (GO:0043068), positive regulation of the apoptotic process (GO:0043065), the extrinsic apoptotic signaling pathway (GO:0097191), and regulation of extrinsic apoptotic signaling (GO:2001236). Concurrent activation of the prostaglandin metabolic process (GO:0006693) and cholesterol metabolic process (GO:0008203) also suggested that PCP modulated lipid mediators involved in inflammation. Moreover, the continued enrichment of leukocyte chemotaxis (GO:0030595) and chemotaxis (GO:0006935) implied a sustained recruitment of immune cells even at a higher dose.

Overall, these results illustrate a dose-dependent immunomodulatory progression induced by PCP. At a low dose, PCP primarily enhances immune cell migration and related signal transduction. As the concentration increases, it amplifies cytokine-mediated responses and macrophage activity. At the highest dose, PCP further promotes regulated cell death and supports the resolution of inflammation. This dynamic progression underscores PCP’s potential to fine-tune immune responses through transcriptomic reprogramming.

KEGG pathway enrichment analysis provided further support for the dose-dependent immunomodulatory role of PCP. In the low-dose group (MX-P500), DEGs were significantly enriched in key signaling pathways related to cellular stress and early immune activation, including the MAPK (dre04010), mTOR (dre04150), and FoxO (dre04068) signaling pathways. Enrichment in apoptosis (dre04210) and efferocytosis (dre04148) pathways was also observed, indicating the early involvement of programmed cell death and the clearance of apoptotic cells. Furthermore, the activation of lysosome (dre04142) and cell adhesion molecule (dre04514) pathways indicates enhanced immune surveillance, phagolysosomal function, and intercellular communication during initial immune activation.

In the medium-dose group (MX-P1000), DEGs were enriched in energy metabolism and glycan biosynthesis pathways. Specifically, oxidative phosphorylation (dre00190), glycolysis/gluconeogenesis (dre00010), and N-glycan biosynthesis (dre00510) were upregulated, reflecting increased bioenergetic demands and protein glycosylation required for immune cell activation. Simultaneously, enrichment of the MAPK (dre04010), mTOR (dre04150), and Apelin (dre04371) signaling pathways suggests a coordinated activation of immune and stress-adaptive signaling networks that may prime the innate immune response. At high PCP concentrations (MX-P2000), enrichment shifted toward immune-specific signaling pathways, particularly those associated with pattern recognition and inflammatory responses, including the C-type lectin receptor signaling pathway (dre04625), NOD-like receptor signaling pathway (dre04621), RIG-I-like receptor signaling pathway (dre04622), and cytosolic DNA-sensing pathway (dre04623). These are classical innate immune pathways involved in the detection of pathogens and initiation of inflammatory signaling. Continued enrichment in FoxO, MAPK, and mTOR signaling also indicates sustained signal transduction. Additionally, the proteasome pathway (dre03050) and increased efferocytosis point to active protein turnover and clearance of damaged or apoptotic cells.

Raoul Saggini and Alvaro Avivar-Valderas reported that MAPK and mTOR cascades are essential for coordinating immune homeostasis, cell metabolism, and inflammatory responses [56,57]. The MAPK signaling network governs innate immune activation, cell proliferation and cytokine secretion, whose overactivation drives severe inflammation, whereas moderate signaling restoration helps recover suppressed immune function [56]. The mTOR pathway acts as a regulator of neutrophil growth, protein synthesis and metabolic homeostasis, and its dysfunction is associated with immunosuppression [57]. Koffi Kouakou et al. reported that polysaccharides isolated from *Clerodendrum splendens* CSP-AU1 are a potent natural innate immunomodulator which could enhance the phosphorylation levels of multiple key kinases in human PBMC [58]. CSP-AU1 activates immune cells like PBMCs and monocyte/macrophages primarily through the TLR4-mediated signaling pathway. This activation involves key cascades, including the phosphorylation of MAPK family members and mTOR-related kinases, ultimately leading to the stimulation of transcription factors AP-1/NF-κB and the production of cytokines and nitric oxide, contributing to immunomodulatory effects from plant polysaccharides.

In this study, the KEGG enrichment analysis identified MAPK and mTOR signaling pathways as two of the most significant enrichment pathways across low-, medium- and high-dose PCP treatment zebrafish groups. MAPK signaling represents a major downstream cascade triggered by pattern recognition receptors, including RIG-I-like receptors, NOD-like receptors, and C-type lectin receptors [59], all of which were significantly enriched in the high-dose PCP treatment zebrafish groups (MX-P2000). This transcriptomic data provides a coherent mechanistic framework linking upstream receptor-level sensing to coordinated MAPK and mTOR pathway activation in response to PCP, which together could account for the observed restoration of neutrophil numbers and function. The high-dose PCP (MX-P2000) significantly increased neutrophil numbers relative to the model group (Figure 3, Table 1), indicating a pronounced immune-enhancing effect. Consistent with this phenotype, KEGG analysis revealed significant enrichment of multiple pattern recognition receptor pathways, including C-type lectin, NOD-like, RIG-I-like, and cytosolic DNA-sensing pathways, in the MX-P2000 group (Figure 5C). Our data indicate activation of mTOR-linked effectors such as upregulation of *eif4e1c*, *clip1b*, *atp6v1aa*, and downregulation of *ptena*, which is a negative PI3K Akt mTOR regulator. Mechanistically, MAPK pathway kinases can relieve TSC2-mediated inhibition of mTORC1 [60], providing a well described route for MAPK to mTOR cross-talk that couples receptor-driven transcriptional programs to anabolic and translational control. Thus, the concurrent transcriptional signature, comprising MAPK activation, mTOR effector induction, and loss of negative regulators, supports a coordinated MAPK-mTOR signaling axis in PCP-treated zebrafish.

Together, these results demonstrate a progressive shift from general cellular signaling and metabolic adaptation at low and medium doses toward robust immune activation and inflammatory signaling at high PCP concentrations. This pattern aligns with the observed increase in immune cell recruitment and function, particularly neutrophil accumulation, and underscores PCP’s potential to modulate immune responses.

In a previous RNA-Seq study investigating the immunomodulatory activity of PCP in RAW264.7 macrophages, their RNA-Seq analysis revealed that PCP activated classical innate immune pathways, including NF-κB, JAK-STAT, and MAPK signaling, leading to enhanced macrophage cytokine production and effector function [61]. Although macrophages and neutrophils represent distinct components of the innate immune system, our current study observed the same enrichment of the MAPK signaling pathway in neutropenic zebrafish treated with PCP. This cross-species consistency strengthens the translational relevance of our findings and suggests that MAPK-mediated transcriptional reprogramming may be a feature of PCP’s immunomodulatory profile. Transcriptome analysis in that study revealed pathway enrichment in the liver for autophagy, nucleotide metabolism, PPAR signaling, FoxO signaling, and protein processing in the endoplasmic reticulum, while the intestinal transcriptome showed significant enrichment in ribosome biogenesis in eukaryotes. Among these, the FoxO signaling pathway, which is known to play a pivotal role in immune homeostasis and oxidative stress response, was also enriched in our low-dose PCP (MX-P500) and high-dose PCP (MX-P2000) treatment groups, suggesting a shared regulatory target among polysaccharide-based immunomodulators.

**Figure 8:**
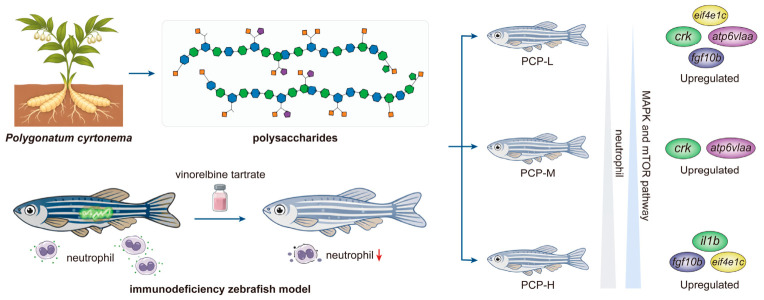
Schematic diagram illustrating the potential mechanisms of immunomodulatory effects exerted by PCP in immunodeficient zebrafish. PCP-L, PCP-M, and PCP-H denote *Polygonatum cyrtonema* polysaccharides administered at concentrations of 500, 1000, and 2000 μg·mL^−1^, respectively. The red downward arrow indicates a reduction in neutrophil counts in the immunodeficient zebrafish model.

Compared to these studies, our transcriptomic data from the low-dose (MX-P500), medium-dose (MX-P1000) and high-dose (MX-P2000) PCP treatment groups revealed a broader and more intensive enrichment of immune-related signaling pathways. These results point toward a more robust activation of immune function, particularly in the context of neutrophil-mediated immunity. Moreover, RT-qPCR validation of key differentially expressed genes (DEGs) within these pathways, including *il1b*, *crk*, *fgf10b*, *atp6v1aa*, and *eif4e1c*, strongly supported the reliability of the RNA-Seq findings (Figure 7 and Figure 8).

Although our transcriptomic analysis identified significant enrichment of the MAPK and mTOR signaling pathways following PCP treatment, the limitation of relying solely on transcriptomic data is that mRNA abundance does not always correlate with protein activity. Both MAPK and mTOR pathways are predominantly regulated through post-translational modifications, especially protein phosphorylation events that determine pathway activation states [62]. Without assessing the phosphorylation of key effectors for the MAPK or mTOR pathway, the mechanistic interpretation remains incomplete. Despite these limitations, the transcriptomic findings still provide a meaningful starting point for hypothesizing the signaling mechanisms through which PCP enhances neutrophil-mediated immunity. Future studies incorporating phosphorylation assays, inhibitor-based functional experiments, and possibly single-cell signaling analyses will be essential to confirm the direct involvement of MAPK and mTOR pathways in mediating the immunomodulatory effects of PCP.

The zebrafish model offers unique translational advantages for immunomodulatory research due to the high evolutionary conservation of innate immune components and signaling pathways between zebrafish and mammals. Given the limited number of studies investigating immunomodulatory effects of natural polysaccharides in genetically modified zebrafish models, the present work provides novel insights into PCP-mediated immune recovery in *Tg (mpx:GFP)* neutropenic zebrafish. This study demonstrates that PCP activates conserved MAPK and mTOR signaling pathways, establishes regulators of neutrophil proliferation, survival, metabolism, and inflammatory responses, and concomitantly modulates the expression of key immune regulatory genes. The activation of these pathways by PCP in immunodeficient zebrafish therefore suggests that similar molecular mechanisms may underlie PCP-mediated immune enhancement in higher vertebrates. Our findings provide mechanistic and phenotypic evidence that PCP modulates conserved immune signaling networks, supporting its further evaluation in mammalian systems and its potential development as a natural immunomodulatory agent for clinical applications.

## 5. Conclusions

In this study, neutrophil phenotypic assessment combined with transcriptomic analysis was applied to investigate the immunomodulatory effects of PCP in an immunodeficient zebrafish model. PCP was characterized as a fructan-rich polysaccharide with high fructose content which promoted neutrophil recovery in a dose-dependent manner. Transcriptomic and functional analyses indicated that its effects were mainly associated with the regulation of MAPK and mTOR signaling pathways. Within the tested concentrations (500, 1000 and 2000 μg/mL), the highest immunostimulatory response was observed at 2000 μg/mL, indicating an effective concentration under the present experimental conditions. These findings may provide a reference for future dose optimization and translational studies and support the immunoregulatory role of PCP and its potential as a natural immunomodulatory agent.

## Figures and Tables

**Table 1:** Primer sequences used for quantitative real-time PCR (qRT-PCR) analysis in this study.

Primer Name	Primer Sequence	NCBI Accession Number
*β-actin* Fw	ATGGATGAGGAAATCGCTGC	NM_131031.2
*β-actin* Rv	TGGAGGGGAAAACAGCACGA	
*il1b* Fw	GAAAGCAGAGGAACTTAACC	NM_212844.2
*il1b* Rv	TAAACAGCACCGTCTGTCTC	
*crk* Fw	TGGCCGGAAATTTTGATTCG	NM_001003628.2
*crk* Rv	ACTCCATGCCTCTGCCCTTG	
*fgf10b* Fw	GTGCGCCAGAGGAGACTCTT	NM_001045858.1
*fgf10b* Rv	TACTGTACGGGTCGTCTTCG	
*atp6v1aa* Fw	CTGCCTAAGATCCGAGATGA	NM_201135.2
*atp6v1aa* Rv	TAGCTGCACCTGCCATGCTG	
*eif4e1c* Fw	AAACTGAAGAAGTCCGCTCT	NM_001017851.2
*eif4e1c* Rv	TACCAGAGGGCCCATCTGTT	

**Table 2:** Statistical analysis of neutrophil numbers in the caudal vein of *Tg (mpx:GFP)* transgenic zebrafish in each group. Data are expressed as mean ± standard error of the mean (SEM), *n* = 10 per group. Statistical significance was analyzed by one-way ANOVA followed by Dunnett’s multiple comparison test. * *p* < 0.05, *** *p* < 0.001 versus the model control group.

Group	Neutrophil Counts
Normal control	68.1 ± 3.50 ***
Model control	39.9 ± 2.66
Positive control	49.7 ± 2.84
500 µg/mL PCP	42.1 ± 2.40
1000 µg/mL PCP	45.2 ± 2.09
2000 µg/mL PCP	50.2 ± 2.92 *

**Table 3:** Summary statistics of sequencing read quality filtering. MX represents the vinorelbine-induced model control group; P500, P1000, and P2000 represent the model zebrafish treated with 500, 1000, and 2000 μg/mL *Polygonatum cyrtonema* polysaccharides (PCP), respectively. Abbreviations: Q20/Q30, percentages of bases with phred quality scores ≥20 and ≥30, respectively.

Sample	Total Raw Reads (M)	Total Clean Reads (M)	Total Clean Bases (M)	Clean Reads Q_20_ (%)	Clean Reads Q_30_ (%)	Clean Reads Ratio (%)
MX_1	47.19	44.77	6.72	97.72	92.64	94.88
MX_2	48.93	45.13	6.77	97.82	92.92	92.23
MX_3	48.93	45.30	6.80	97.75	92.69	92.58
P500_1	47.19	45.47	6.82	97.43	91.76	96.37
P500_2	47.19	44.81	6.72	97.67	92.45	94.96
P500_3	47.19	44.61	6.69	97.69	92.52	94.55
P1000_1	47.19	45.15	6.77	97.62	92.30	95.68
P1000_2	47.19	44.52	6.68	97.72	92.61	94.34
P1000_3	47.19	43.98	6.60	97.66	92.44	93.21
P2000_1	47.19	44.55	6.68	97.73	92.64	94.41
P2000_2	47.19	44.09	6.61	97.67	92.46	93.44
P2000_3	47.19	44.14	6.62	97.70	92.59	93.54

## Data Availability

The datasets utilized in this study are publicly available in online repositories. The raw sequencing data are available in the NCBI SRA database under BioProject PRJNA1256770 (https://www.ncbi.nlm.nih.gov/bioproject/PRJNA1256770, accessed on 29 April 2025).

## Supplementary Materials

The following supporting information can be downloaded at https://www.mdpi.com/article/10.3390/cimb48050494/s1.
