# Supplementary material for: Immune-Enhancing Effects of Polygonatum cyrtonema Polysaccharides in Immunodeficient Zebrafish
Source: Curr Issues Mol Biol. 2026 May 9;48(5):494. doi: 10.3390/cimb48050494 (PMC13204381; doi:10.3390/cimb48050494)
Supplement: Supplementary file 1 [file cimb-48-00494-s001.zip › cimb-4279906-supplementary.pdf]

Supplementary Table S1. Minimum Toxic Concentration (MTC) of *Polygonatum cyrtonema* Polysaccharides in Zebrafish

Group	Concentration of PCP(μg/mL)	200μg/mL vinorelbine tartrate (nL)	Mortality (Number of dead Zebrafish /Total)	Mortality Rate (%)	Phenotypic Observations
Normal control	0	0	0/30	0	No obvious abnormality
Model control	0	10	0/30	0	No obvious abnormality
PCP	31.2	10	0/30	0	Normal, comparable to normal control
	62.5	10	0/30	0	Normal, comparable to normal control
	125	10	0/30	0	Normal, comparable to normal control
	250	10	0/30	0	Normal, comparable to normal control
	500	10	0/30	0	Normal, comparable to normal control
	1000	10	0/30	0	Normal, comparable to normal control
	2000	10	0/30	0	Normal, comparable to normal control

Supplementary Table S2. DEGs in the MAPK and mTOR signaling pathways

Group	KEGG Pathway	GENE ID	GENE Symbol	Log_2fc
MX_500	MAPK signaling pathway	565241	ppp3ccb	1.00241
MX_500	MAPK signaling pathway	405770	il1b	1.27433
MX_500	MAPK signaling pathway	406304	gadd45ba	-1.59456
MX_500	MAPK signaling pathway	337670	rps6kal	-1.12155
MX_500	MAPK signaling pathway	497646	gadd45bb	-1.08168
MX_500	MAPK signaling pathway	570238	fosaa	-1.09947
MX_500	MAPK signaling pathway	394198	fosab	-2.7782
MX_500	MAPK signaling pathway	493609	grb2a	-1.20101
MX_500	MAPK signaling pathway	431763	gadd45ab	-1.3346
MX_500	MAPK signaling pathway	58130	tradd	-4.8899
MX_500	MAPK signaling pathway	324215	igf2b	-1.04053
MX_500	MAPK signaling pathway	445234	crk	2.79645
MX_500	MAPK signaling pathway	751792	fgf10b	1.95201
MX_1000	MAPK signaling pathway	570238	fosaa	-1.08938
MX_1000	MAPK signaling pathway	394198	fosab	-1.15079
MX_1000	MAPK signaling pathway	559630	fgf20a	1.61014
MX_1000	MAPK signaling pathway	431763	gadd45ab	-1.04431
MX_1000	MAPK signaling pathway	565241	ppp3ccb	-2.34078

MX_1000	MAPK signaling pathway	324215	igf2bp3	-1.17436
MX_1000	MAPK signaling pathway	445234	crk	2.67712
MX_2000	MAPK signaling pathway	405770	il1b	2.15885
MX_2000	MAPK signaling pathway	140621	casp3a	-1.2282
MX_2000	MAPK signaling pathway	431763	gadd45ab	-1.24445
MX_2000	MAPK signaling pathway	58130	tradd	-1.48336
MX_2000	MAPK signaling pathway	30262	ins	3.02129
MX_2000	MAPK signaling pathway	493609	grb2a	-1.29307
MX_2000	MAPK signaling pathway	562552	zgc	2.45478
MX_2000	MAPK signaling pathway	751792	fgf10b	1.86551
MX_500	mTOR signaling pathway	100537790	clip1b	1.71551
MX_500	mTOR signaling pathway	378866	ddit4	-1.15434
MX_500	mTOR signaling pathway	550549	eif4e1c	1.47516
MX_500	mTOR signaling pathway	337670	rps6kal	-1.12155
MX_500	mTOR signaling pathway	394110	atp6v1aa	1.44535
MX_500	mTOR signaling pathway	493609	grb2a	-1.20101
MX_500	mTOR signaling pathway	794088	ptena	-1.1298
MX_500	mTOR signaling pathway	798071	sec13	-1.35395
MX_1000	mTOR signaling pathway	100537790	clip1b	1.65752
MX_1000	mTOR signaling pathway	550549	eif4e1c	1.26366
MX_1000	mTOR signaling pathway	3012	wnt2	-2.12757
MX_1000	mTOR signaling pathway	394110	atp6v1aa	1.44385
MX_1000	mTOR signaling pathway	562242	rps6kb1a	-1.417
MX_2000	mTOR signaling pathway	550549	eif4e1c	1.69702
MX_2000	mTOR signaling pathway	794088	ptena	-1.27776
MX_2000	mTOR signaling pathway	30262	ins	3.02129
MX_2000	mTOR signaling pathway	493609	grb2a	-1.29307
MX_2000	mTOR signaling pathway	30127	wnt2	-1.30769
MX_2000	mTOR signaling pathway	100537790	clip1b	1.6068
